# Mobile phone use and risk of acoustic neuroma: results of the interphone case–control study in five North European countries

**DOI:** 10.1038/sj.bjc.6603070

**Published:** 2006-03-28

**Authors:** L Hardell, K Hansson Mild

**Affiliations:** 1 Department of Oncology, University Hospital, Örebro, SE-701 85 Sweden; 2 Department of Natural Sciences, Örebro University, Örebro, SE-701 82 Sweden; 3 National Institute for Working Life, Umeå, SE-907 13 Sweden

**Sir**,

Recently a significantly increased risk for acoustic neuroma was reported for 10 years or more use of mobile phone on the same side of the head as the tumour developed (Schoemaker *et al*, 2005). Thus, our previous reports of such an association were confirmed (Hardell *et al*, 2002, 2003a, 2003b, 2005a). However, the authors write that there is no substantial risk in the first decade after starting of mobile phone use, a statement that has echoed in news media as the main result and has little significance in long-term carcinogenesis.

We are surprised that the authors claim that our first study (Hardell *et al*, 2002, 2003a, 2003b) has been ‘heavily criticised for methodological limitations’. They give reference to five short comments or reports, two published in 2000 and 2001 (Rothman, 2000, 2001), thus even before our publication! The report by Boice and McLaughlin (2002) has never been published in a prereview journal and they are employed at an institute that has been linked to Motorola (Hardell, 2004). The other two references include authors of the Interphone study, thus merely themselves. No information is given in the paper (Schoemaker *et al*, 2005) regarding our ‘methodological limitations’, so we compare standard epidemiological methods in the Interphone study and our studies (same methods were used in our two studies).

Controls were in the Interphone study recruited from general practioners' lists (UK), whereas all controls in the Hardell *et al* studies were selected from the population register.

Recall and observation bias was probable in the Interphone study since interviews were computer based and conducted at the subject's home, the hospital or another place. Thus, the situation might have been stressful for the interviewed person; the interviewer knew if it was a case or a control that was interviewed, the interview place was not standardised and a large number of interviewers must have been involved (numbers not presented). Furthermore, we are not told how controls were selected living in vast areas such as Northern Sweden; only from the largest cities? At least in the Norwegian part of the study some of the controls were recruited by phone, thus giving potential for selection bias as to use of cellular telephones. Did the interviewers travel long distances or were controls with uncomfortable addresses disregarded?

The study group had knowledge during the full process until statistical analysis if it was a case or a control. In contrast, the Hardell *et al* studies used standard methods with questionnaires sent to the homes of cases and controls and if necessary supplemented over the phone. Assessment of exposure was performed blinded as to case or control status, as well as coding and registration of exposure for statistical analysis in our studies.

As we have previously commented (Hardell and Hansson Mild 2005b) on the Swedish part (Lönn *et al*, 2004) of this Interphone study the numbers in the different tables are not easy to follow. Schoemaker *et al* (2005) present in Table 2 numbers of subjects with ⩾10 years lifetime use of mobile phone; 31 cases and 131 controls. However, in Table 4 analysing mobile phone use and laterality of tumour the corresponding numbers are 35 cases and 145 controls. Moreover, numbers of ‘unexposed’ are not consistent in these two tables.

We assessed also use of cordless phones in contrast to the Interphone study. As we have discussed elsewhere (Hardell *et al*, 2006), such microwave exposure should also be included in this type of studies. As reference category we used subjects with no report of use of cellular or cordless phones. However, in contrast to us the Interphone study had no constant unexposed group for mobile phone use and the ‘unexposed’ category would furthermore include cordless phone users, thus diluting the risk towards unity.

The authors stated that the studies were partly financed by the telecom industry. According to IARC the funding from industry was 3.5 million Euros, and from the European Union, 3.85 million Euros (E Cardis, personal communication). The contract stipulated that the industry has the right to be informed about the results a maximum of 7 days before the publication (IARC, 2005).

Receiving grants from industry is by the International Committee of Medical Journal Editor regarded as ‘the most important conflicts of interest’. In a review of health studies on environmental tobacco smoke the rate ratio of a paper with at least one author with industry associations reaching an industry favourable conclusion was 3.2, 95% CI 1.4–7.5 (Garne *et al*, 2005). Could this explain the almost excuse of the own results, see last paragraph in the paper, and the scientifically unfounded criticism of our studies by the Interphone study authors?
